# Supplementary material for: Daily Caffeine Consumption May Increase the Risk of Acute Kidney Injury Related to Platinum-Salt Chemotherapy in Thoracic Cancer Patients: A Translational Study
Source: Nutrients. 2024 Mar 19;16(6):889. doi: 10.3390/nu16060889 (PMC10975520; doi:10.3390/nu16060889)
Supplement: Supplementary file 1 [file nutrients-16-00889-s001.zip › nutrients-2916395-supplementary.pdf]

Supplementary Table S1. Conversion tables: standard volumes and caffeine containers.

**Evaluation of Quantities: Standardization of Containers**		
**Containers**	**Usual Volumes (mL)**	**Mean Effective Volume (mL)**
Bowl	250–350	300
Large cup (tea cup, hot chocolate cup, mug)	200–300	250
Small cup (coffee cup)	90–120	100
Can	330	330
Tall glass (fruit juice glass)	250–280	265
Standard glass (mustard glass)	150–200	175
Vending machine cup	150–200	175

**Sources of Caffeine**	
**Food Items**	**Average Caffeine Content (mg/100 mL or mg/100 g)**
Decaffeinated coffee	2
Instant coffee	43
Filtered coffee	76
Espresso	133, i.e., 40 mg for one espresso (30 mL)
Brewed tea	26.6
Iced tea	12
Cola beverages (regular, diet)	12
Dark chocolate	64.5
Milk chocolate	25

**Caffeine content in Different Containers (in mg per container)**							
**Food Items**	**Bowl (300 mL)**	**Large Cup (250 mL)**	**Small Cup (100 mL)**	**Can (330 mL)**	**Tall Glass (265 mL)**	**Standard Glass (175 mL)**	**Venting Machine Cup (175 mL)**
Decaffeinated coffee	6	5	2	/	5.3	3.5	3.5
Instant coffee	129	107.5	43	/	114	75.3	75.3
Filtered coffee	228	190	76	/	201.4	133	133
Brewed tea	79.8	66.5	26.6	/	70.5	46.6	46.6
Iced tea	36	30	12	39.6	31.8	21	21
Cola beverages	/	/	/	39.6	31.8	21	21
Espresso	133 mg per 100 mL, i.e., 40 mg per 1 espresso (30 mL)						
**Other Food Items**	1 square (10 g)				1 bar (25 g)		
Dark chocolate	6.5				16		
Milk chocolate	2.5				6.3		
